# Bone marrow mesenchymal stem cell–derived exosomal miR‐206 promotes osteoblast proliferation and differentiation in osteoarthritis by reducing Elf3

**DOI:** 10.1111/jcmm.16654

**Published:** 2021-06-23

**Authors:** Yijiang Huang, Xiumeng Zhang, Jingdi Zhan, Zijiang Yan, Daosen Chen, Xinghe Xue, Xiaoyun Pan

**Affiliations:** ^1^ Department of Orthopaedic The Second Affiliated Hospital and Yuying Children's Hospital of Wenzhou Medical University Wenzhou China; ^2^ Key Laboratory of Orthopaedics of Zhejiang Province Wenzhou China

**Keywords:** E74‐like factor 3, exosome, microRNA‐206, osteoarthritis, osteoblasts, proliferation and differentiation

## Abstract

MicroRNAs (miRNAs) serve as gene silencers involved in essential cell functions. The role of miR‐206 and E74‐like factor 3 (Elf3) has been identified in osteoarthritis (OA), while the effect of exosomal miR‐206 from bone marrow mesenchymal stem cells (BMSCs) in OA remains largely unknown. Thus, we aim to explore the role of exosomal miR‐206 from BMSCs in OA with the involvement of Elf3. BMSCs and BMSC‐derived exosomes (BMSC‐exos) were obtained and identified. OA mouse models were constructed by anterior cruciate ligament transection and then treated with BMSC‐exos or BMSC‐exos containing miR‐206 mimic/inhibitor. The expression of miR‐206, Elf3, inflammatory factors, osteocalcin (OCN) and bone morphogenetic protein 2 (BMP2) in mouse femoral tissues was assessed. The pathological changes in mouse femur tissues were observed. The mouse osteoblasts were identified and treated with untransfected or transfected BMSC‐exos, and then, the expression of miR‐206, Elf3, OCN and BMP2 was determined. The alkaline phosphatase (ALP) activity, calcium deposition level, OCN secretion, proliferation, apoptosis and cell cycle arrest in osteoblasts were measured. MiR‐206 was down‐regulated while Elf3 was up‐regulated in OA animal and cellular models. Exosomal miR‐206 ameliorated inflammation and increased expression of OCN and BMP2 in mouse femoral tissues. Moreover, exosomal miR‐206 promoted ALP activity, calcium deposition level, OCN secretion and proliferation and inhibited apoptosis in OA osteoblasts. Overexpressed Elf3 reversed miR‐206 up‐regulation‐induced effects on OA osteoblasts. BMSC‐derived exosomal miR‐206 promotes proliferation and differentiation of osteoblasts in OA by reducing Elf3. Our research may provide novel targets for OA treatment.

## 1 INTRODUCTION

Osteoarthritis (OA) is the most common form of arthritis and a leading cause of joint pain and disability.^1^ It is estimated that up to 240 million people suffer from OA worldwide. The prevalence of OA is expected to increase due to the increasing ageing population, sedentary behaviour and lack of physical activity.^2^ Pathological changes in OA joints contain lost and destructed articular cartilage, thickened subchondral bone, formed osteophytes, inflammation of synovium, degenerated ligaments and menisci of the knee and hypertrophy of the joint capsule.^3^ OA refers to an enormous health and economic burden on both individual and society. There are no proven effective therapeutic strategies to prevent, decelerate, cease or reverse the OA development. Currently, OA management is mostly palliative and focuses on pain attenuation.^4^ Thus, candidate biomarkers of OA are needed to be explored.

Bone marrow mesenchymal stem cells (BMSCs) are non‐haematopoietic stem cells in bone marrow with the ability of self‐renewal, pronounced proliferation and differentiation into various types of other cells.^5^ Exosomes are small (diameter of 30‐100 nm) membrane vesicles secreted by various cell types.^6^ MSC‐derived exosomes have a wide range of therapeutic effects, such as acute myocardial infarction ^7^ and pulmonary fibrosis.^8^ Meanwhile, exosomes have an effect on joints and bones. For example, exosomes released from BMSCs (BMSC‐exos) have been revealed to suppress chondrocyte apoptosis induced by mitochondrial dysfunction ^9^ and ameliorate pain in subchondral bone in lumbar facet joint OA.^10^ Moreover, BMSC‐exos can effectively promote cartilage repair and extracellular matrix synthesis in OA rats and reduce knee joint pain.^10^, ^11^ MicroRNAs (miRNAs) are small non‐coding RNAs that suppress gene expression via binding the 3′‐untranslated region (3′‐UTR) and influencing mRNA stability or interfering with protein translation.^12^ A large number of exosomes carry miRNA, including miR‐1, miR‐133a, miR‐133b, miR‐206 and miR‐486.^13^ BMSCs can transmit miR‐206,^14^ and miR‐206 is involved in the process of OA.^15^ MiR‐335‐5p has been revealed to relieve chondrocyte inflammation in OA,^16^ and miR‐204 and miR‐211 have been reported to protect from OA progression,^17^ and miR‐206 is required for OA development through its effect on apoptosis and autophagy of articular chondrocytes.^18^ Another study has suggested that miR‐206 functioned as a long non‐coding RNA FOXD2‐AS1 sponge to regulate chondrocyte proliferation in OA.^19^ Thus, we speculated that miR‐206 may play a critical role during OA development. Exosomes carrying miRNAs have been identified to mediate intercellular signal transduction and affect the biological function of donor cells to the receipt cells in diseases.^20^ It has been validated that BMSC‐derived exosomal miR‐29b‐3p prevented hypoxic‐ischaemic injury,^21^ and miR‐206 has been verified to modulate the effect of exosomes derived from KLF3‐AS1‐overexpressing‐MSCs in OA.^15^ Nevertheless, the role of BMSC‐derived exosomal miR‐206 in OA remains rarely explored. The E‐74‐like factor 3 (Elf3) belongs to the Elf subfamily with effect on epithelial cell differentiation and function, and it has been demonstrated to induce cartilage degradation in a model of post‐traumatic OA.^22^ We performed this study to investigate the impact of BMSC‐derived exosomal miR‐206 on the progression of OA, and we assumed that exosomal miR‐206 from BMSCs may regulate osteoblast differentiation by targeting Elf3, thus affecting the OA development.

## 2 MATERIALS AND METHODS

### 2.1 Ethics statement

Animal experiments were strictly in accordance with the Guide to the Management and Use of Laboratory Animals issued by the National Institutes of Health. The protocol of animal experiments was approved by the Institutional Animal Care and Use Committee of The Second Affiliated Hospital and Yuying Children's Hospital of Wenzhou Medical University.

### 2.2 Experimental animals

Adult pathogen‐free C57BL/6 mice (ageing 9 w and weighing 28‐24 g) and 4‐week‐old C57BL/6 mice that obtained from the Experimental Animal Center of Wenzhou Medical University (Zhejiang, China) were fed with well ventilation, 12‐h day/night cycle and temperature at 20‐25°C.

### 2.3 Isolation, culture and identification of BMSCs

Double tibia and femur were harvested from 5 C57BL/6 mice ageing 4 w, and the muscles were removed. The both ends of the bones were cut off to expose the marrow cavity, and the marrow was washed out by Dulbecco's modified Eagle medium (DMEM)/F12; thus, the mixed cell suspension was obtained. The suspension was filtered and centrifuged at 1000 r/min for 5 minutes with the supernatant discarded to acquire sediments containing BMSCs, which were then incubated in DMEM/F12 containing 10% foetal bovine serum (FBS) and 1% penicillin‐streptomycin (P/S) at 37°C with 5% CO_2_. The total medium was changed after 2 days, and from then on, the medium was replaced every 2‐3 days. The morphology and growth of cells were observed under an inverted microscope. Cells were passaged at 80% cell confluence. Then, cells were passaged every 3‐5 days.

P4 BMSCs (~1 × 10^6^ cells) were loaded into Eppendorf (EP) tubes, and cells in each tube were resuspended using 100 μL phosphate‐buffered solution (PBS). One tube containing empty cells without fluorescent antibody labelling was set as the negative control (NC). Three tubes were respectively set as the homotype controls of phycoerythrin (PE), fluorescein isothiocyanate (FITC) and PerCP‐Cy5.5. Other tubes were severally added with 1.5 μL CD34‐FITC, CD29‐PE, CD45‐FITC and CD90‐PerCP‐Cy5.5 flow antibodies (BD Biosciences) and incubated for more than 40 minutes without light exposure. Centrifuged with the supernatant discarded, cells were resuspended with 40 or 60 μL PBS and then detected by an Amnis ImageStream X Mark II flow cytometer (Merck KGaA). The adipogenic and osteogenic inductions were conducted in line with the instructions of adipogenic and osteogenic differentiation medium (Gibco Company). Oil red O staining and alizarin red staining were performed after the induction, and the staining was observed under a microscope.

### 2.4 Transfection and grouping of BMSCs

P3 BMSCs were seeded onto 12‐well plates and transfected based on directions of Lipofectamine 2000 reagent (Invitrogen Inc) when cell confluence reached 80%. MiR‐206 mimic, miR‐206 inhibitor and their NCs were acquired from GenePharma Co., Ltd.. BMSCs were divided into 4 groups: the blank group (no treatment), the NC group (transfection of irrelevant control), the miR‐206 mimic group (transfection of miR‐206 mimic) and the miR‐206 inhibitor group (transfection of miR‐206 inhibitor). Cells in each group that had been transfected for 72 hours were used in subsequent experiments.

### 2.5 Extraction and identification of BMSC‐exos

P4 BMSCs were trypsinized, resuspended, cultured for 48 hours and centrifuged at 1500 *g* for 15 minutes. Supernatant was incubated with 50% ExoQuick‐TC exosome precipitation liquid (ExoQuick‐TC Kit; System Biosciences) at 4°C overnight and then centrifuged at 10 000 *g* for 30 minutes. The sediment was centrifuged at 10 000 *g* for 2 hours and mixed with PBS to obtain relatively pure exosomes. The exosome sediments at the bottom of tubes were light brown or white.^23^

Transmission electron microscope (TEM) imaging of the exosomes: 10 μL exosome samples were loaded into new EP tubes and added onto a loading copper network that had been rinsed by 100% ethanol. The copper network was supplemented with a drip of 1% uranyl acetate for 30 seconds, and the exosomes were observed through a TEM (Hitachi, Ltd.).

Nanoparticle tracking analysis: The exosomes were diluted and put into the NanoSight NS300 (Malvern Instrument). The motion trail of the particles was observed, and the diameter and concentration of the particles were calculated.

Identification of exosomes: Protein concentration of exosomes was determined, and 10% separation gel and 5% spacer gel were prepared. The proteins were denatured, loaded and transferred onto membranes and then blocked with 5% bovine serum albumin (BSA) for 1 hours and incubated with primary antibodies CD63 and Alix at 4°C overnight. Next, the proteins were incubated with secondary antibody for 1 hour and developed.

### 2.6 Establishment of OA mouse models

Mice were anaesthetized by intraperitoneal injection of 3% pentobarbital sodium (1 mL/kg). The mouse right knee joint was exposed by the medial para‐patellar approach. The patella was dislocated, and the knee was completely flexed. The anterior cruciate ligament was then cut off by a micro‐scissor, and the transection was confirmed with the anterior drawer test. The joint surface was washed with normal saline after the surgery, and the Vicryl 4‐0 (Ethicon), absorbable suture and mono‐filament 4‐0 Nylon threads (Ethicon) were used for suture. Buprenorphine hydrochloride (0.1 mg/kg) (Reckitt & Colman Products Ltd., Hull, England) was utilized as a post‐operative analgesic.^24^ Clinical OA scoring ranged from 0 to 4 points referring to a publication.^25^ The OA score of mice was calculated as the sum of points of each limb, and the highest score was 16 points.

### 2.7 Treatment of collagen‐induced arthritis (CIA)

Treatment was initiated after the onset of disease (28 days after the ﬁrst immunization) when the mice were successfully modelled (arthritis score ≥2 points). The articular cavity was injected with 100 μL exosomes twice/w (10^11^ exosome particles/mL were injected into 10 knee joints from 5 mice).

Mice were classified into 6 groups: The sham group (patellar was dislocated but anterior cruciate ligament was not cut off, n = 10), the OA group (mice were subjected to CIA modelling, n = 15), the Exo group (articular cavity of modelled mice was injected with non‐transfected BMSC‐exos, n = 10), the NC‐Exo group (articular cavity of modelled mice was injected with BMSC‐exos that had been transfected with miR‐206 mimic NC, n = 10), the miR‐206 mimic‐Exo group (articular cavity of modelled mice was injected with BMSC‐exos that had been transfected with miR‐206 mimic, n = 10) and the miR‐206 inhibitor‐Exo group (articular cavity of modelled mice was injected with BMSC‐exos that had been transfected with miR‐206 inhibitor, n = 10).

### 2.8 Collection of mouse bone tissues

Eight weeks after modelling and treatment with exosomes, the mice were anaesthetized and blood was collected from the heart, and then, the mice were killed with their femurs isolated. The left femur was preserved at −80°C for enzyme‐linked immunosorbent assay (ELISA), reverse transcription quantitative polymerase chain reaction (RT‐qPCR) and Western blot analysis; the right femur was fixed with 10% formalin solution for haematoxylin‐eosin (HE) staining, terminal deoxynucleotidyl transferase–mediated deoxyuridine triphosphate nick end‐labelling (TUNEL) staining and micro‐computerized tomography (CT) examination.

### 2.9 ELISA

Mouse heart blood was centrifuged at 4°C and 3000 r/min for 10 minutes, and the serum was collected to measure the levels of osteocalcin (OCN) and alkaline phosphatase (ALP) based on the manufactures’ information of ELISA kits (Bio‐swamp). The bone tissues were homogenized and centrifuged at 2500‐3000 rpm for 10 minutes with the supernatant collected. The levels of tumour necrosis factor‐α (TNF‐α), interleukin (IL)‐6 and IL‐1β were determined using ELISA kits (Bio‐swamp).

### 2.10 HE staining

The bone tissues were decalcified, paraffin‐embedded and sectioned. Sections were marked by HE staining histological score. The degrees of synovial inflammation or cartilage injury were measured according to description in a previous study.^26^

### 2.11 TUNEL staining

TUNEL staining was performed based on the instructions of kits obtained from Roche Ltd. Mice femoral head was sectioned, dewaxed, hydrated and treated with 3% H_2_O_2_ for 10 minutes and then digested by 20 μg/mL proteinase K at 37°C for 20 minutes and blocked with 5% BSA (25 μL/section) at 37°C for 30 minutes. With the blocking buffer removed, each section was supplemented with 25 μL mixture of terminal deoxynucleotidyl transferase and digoxin‐deoxyuridine triphosphate (1:9) for 60‐minutes incubation and peroxidase reagent was added for 40‐minutes incubation. The sections were developed using diaminobenzidine, counterstained by haematoxylin, dehydrated, permeabilized, sealed and observed under a microscope. Three fields of view were selected, and the positive cell percentage in 100 cells of each field was calculated.

### 2.12 Micro‐CT examination

Mouse right femur was fixed in 80% ethanol with light avoidance for 3 days and scanned by micro‐CT (eXplore Locus SP, General Electric Company). The samples were performed with three‐dimensional reconstruction, and then, the bone mineral density (BMD), bone volume/total volume (BV/TV), trabecular space (Tb. Sp) and trabecular number (Tb.N) were analysed.

### 2.13 Isolation and extraction of mouse osteoblasts

Knee joint samples from 5 remaining mice in the OA group were rinsed by balanced salt solution containing 100 U/mL P/S, and the trabecula was soaked in D‐hanks solution. With the remaining fat and blood removed, the trabecula was chopped and incubated with DMEM containing 10% FBS. The bone tissue blocks were discarded after cells covered the bottom of the culture bottle, and the osteoblasts were extracted by trypsinization and centrifugation. The medium was changed after 24 hours, and subsequently, the medium was replaced every 3 days. Passage experiment was conducted after the cell confluence reached 80%. ALP staining was performed by Gomori's calcium cobalt method, and the mineralized nodule staining was conducted by alizarin red method; thus, the osteoblasts were identified. P3 cells were used in experiment.

### 2.14 Grouping of mouse osteoblasts

BMSC‐exos were resuspended by DMEM containing 10% FBS and co‐cultured with OA mouse osteoblasts for 3 days. The obtained OA mouse osteoblasts were classified into 9 groups: the OA group (OA mouse osteoblasts), the Exo group (osteoblasts were co‐cultured with non‐transfected BMSC‐exos), the mimic NC‐Exo group (osteoblasts were co‐cultured with miR‐206 mimic NC‐transfected BMSC‐exos), the miR‐206 mimic‐Exo group (osteoblasts were co‐cultured with miR‐206 mimic‐transfected BMSC‐exos), the mimic NC group (osteoblasts were transfected with miR‐206 mimic NC), the miR‐206 mimic group (osteoblasts were transfected with miR‐206 mimic), the short hairpin RNA (sh)‐NC group (osteoblasts were transfected with NC of sh‐Elf3), the sh‐Elf3 group (osteoblasts were transfected with sh‐Elf3) and the miR‐206 mimic +overexpressed (oe)‐Elf3 group (osteoblasts were transfected with miR‐206 mimic and overexpressed Elf3 vector).

### 2.15 ALP and alizarin red staining

The ALP and alizarin red staining were performed as previously described, and the absorbance at 562 nm was measured.^27^

### 2.16 Measurement of OCN secretion

Osteoblasts were seeded for 3‐days culture, and the medium was performed with lyophilization and OCN labelling. A radio‐immunity γ counter (Hefuguangdian Instrument Co., Ltd.) was used to detect the radioactivity; thus, the OCN secretion was measured.

### 2.17 3‐(4,5‐dimethyl‐2‐thiazolyl)‐2,5‐diphenyl‐2‐H‐tetrazolium bromide (MTT) assay

P3 osteoblasts were seeded onto 48‐well plates at 5 × 10^3^ cells/well and incubated at 37°C with 5% CO_2_. MTT solution (20 μL, 5 g/L) was added onto each well at the 24th, 48th and 72nd hours of the incubation. After 4‐hours incubation, the supernatant was discarded and each well was appended with 150 μL dimethyl sulphoxide solution. A microplate reader (LabSystems Multiskan MS, Labsystems) was used to analyse the absorbance at 490 nm.

### 2.18 Flow cytometry

Cell cycle detection: The osteoblasts were made into single cell suspension (1 × 10^6^ cells/mL), which was centrifuged at 2000 r/min for 5 minutes with the supernatant discarded, fixed with 70% cold ethanol at 4°C overnight, eluted by 1 mL PBS and centrifuged with the supernatant removed. Afterwards, the cells were added with 100 μL RNase A (Sigma) and conducted with 37°C water bath for 30 minutes and then stained with 400 μL propidium iodide (Sigma) at 4°C without light exposure for 30 minutes. A flow cytometer (BD Biosciences) was used for detection.

Cell apoptosis detection: Osteoblasts in each group were collected, and the concentration was adjusted to 1 × 10^6^ cells/mL, and then, 200 μL cells were rinsed with 1 mL pre‐cold PBS twice and centrifuged. Cells were resuspended into 100 μL binding buffer, which were then incubated with 2 μL Annexin‐V‐FITC (20 μg/mL, Sigma) on ice with light avoidance for 15 minutes. The cells were transferred into flow tubes supplemented with 300 μL PBS and examined by a flow cytometer in 30 minutes. Before the detection, each sample was added with 1 μL PI (50 μg/mL, Sigma).

### 2.19 Dual luciferase reporter gene assay

The 3′UTR of Elf3 with the wild‐type (WT) or mutant type (MUT) binding sites of miR‐206 were amplified and inserted into pMIR‐REPORT luciferase reporter vector (Ambion), defined as Elf3‐WT and Elf3‐MUT. The 293T cells were co‐transfected with 200 ng luciferase reporter vectors, 25 ng pRL‐TK (expressing Renilla luciferase as the internal control) and 20 μM miR‐206 mimic or miR‐206 mimic NC by Lipofectamine 2000 reagent. The luciferase report gene detection system (Promega Corporation) was used to analyse the luciferase activity after 24‐hours transfection.

### 2.20 RT‐qPCR

TRIzol kits (Invitrogen) were used to extract the total RNAs from osteoblasts and mouse femur. According to the instructions of iScript cDNA kits (Bio‐Rad Inc.), the RNAs were reversely transcribed into cDNAs. RT‐qPCR was conducted based on directions of SYBR^®^Premix Ex Taq^TM^ kits (Takara Bio, Inc.). Primers are shown in Table 1. U6 and glyceraldehyde phosphate dehydrogenase (GAPDH) were respectively used as the loading control of miR‐206 and Elf3. Data were analysed by 2^−ΔΔCt^ method.

**TABLE 1:** Primer sequence

Gene	Primer sequence (5′‐3′)
miR‐206	Forward: 5′‐GGGTGGAATGTAAGGAAGT‐3′
	Reverse: 5′‐CGTGTCGTGGAGTC‐3′
U6	Forward: 5′‐GCTTCGGCAGCACATATACTAAAAT‐3′
	Reverse: 5′‐CGCTTCACGAATTTGCGTGTCAT‐3′
Elf3	Forward: 5′‐GCCCTCCGTTTCTTACTTCAA‐3′
	Reverse: 5′‐CTCTTCGCACTTCTGCTCCTC‐3′
GAPDH	Forward: 5′‐GGGAAACTGTGGCGTGAT‐3′
	Reverse: 5′‐GAGTGGGTGTCGCTGTTGA‐3′

Note:

miR‐206, microRNA‐206; Elf3, E74‐like factor 3; GAPDH, glyceraldehyde phosphate dehydrogenase.

### 2.21 Western blot analysis

Proteins extracted from osteoblasts and mouse left femur were dissolved in radio‐immunoprecipitation assay buffer containing 50mm Tris‐HCl (pH = 7.4), 150 mmol/L NaCl, 0.1% sodium dodecyl sulphate (SDS), 1% sodium deoxycholate, 1 mmol/L ethylene diamine tetraacetic acid, 1% Triton X‐100 and protease inhibitor. The proteins were extracted at 4°C for 30 minutes. After performed with 10% SDS‐polyacrylamide gel electrophoresis (20 μg/lane), the proteins were transferred onto membranes (Millipore, MA, USA), which were blocked with 5% skim milk and incubated with primary antibodies Elf3 (1:1,000, Abcam Inc.) and GAPDH (1:2000) at 4°C overnight. Next, the membranes were incubated with horseradish peroxidase–conjugated secondary antibody (1:2000, Santa Cruz Biotechnology, Inc.). GAPDH was used as the loading control, and proteins were detected using enhanced chemiluminescent kits (Applygen Technologies). The Image‐Pro Plus 6.0 was used to analyse the protein bands.

### 2.22 Statistical analysis

All data analyses were conducted using SPSS 21.0 software (IBM Corp.). The measurement data were expressed as mean ±standard deviation. The unpaired t test was performed for comparisons between two groups; one‐way analysis of variance (ANOVA) was used for comparisons among multiple groups; and Tukey's post hoc test was used for pairwise comparisons after one‐way ANOVA *P* value <.05 was indicative of statistically significant difference.

## 3 RESULTS

### 3.1 Identification of BMSCs, BMSC‐exos and osteoblasts

**FIGURE 1:**
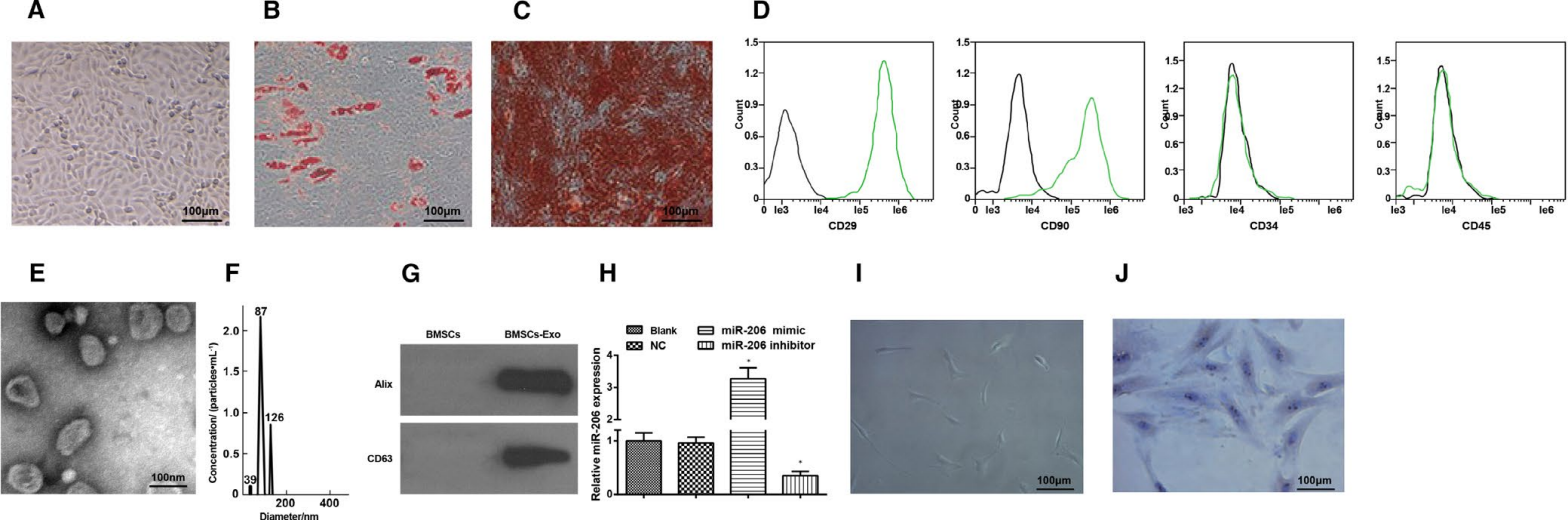
Identification of BMSCs, BMSC‐exos and osteoblasts. A, observation of BMSC morphology; B, BMSC morphology after adipogenic induction; C, BMSC morphology after osteogenic induction; D, BMSC surface antigens were detected by flow cytometry; E, BMSC‐exos were identified by a TEM; F, exosome diameter was measured by NTA; G, expression of CD36 and Alix was detected by Western blot analysis; H, expression of exosomal miR‐206 was assessed using RT‐qPCR; I, observation of osteoblast morphology; J, osteoblasts were identified by ALP staining; N = 3; *, *P* < .05 *vs* the NC group; the measurement data were expressed as mean ±standard deviation, one‐way ANOVA was used for comparisons among multiple groups, and Tukey's post hoc test was used for pairwise comparisons after one‐way ANOVA

Under a microscope, BMSCs were long fusiform and rapidly grew in an obvious swirl shape (Figure 1A). The adipogenic induction was followed by oil red O staining, and it was observed under a microscope that there were orange lipid droplets in cells; the osteogenic induction was followed by alizarin red staining, and obvious red calcium nodule sediments were found (Figure 1B,C). Results of flow cytometry indicated that the BMSC surface biomarkers CD29 and CD90 were both positively expressed while the labelled haematopoietic cell surface biomarkers CD34 and CD45 were both negatively expressed, which were in line with the BMSC phenotype (Figure 1D).

As observed through a TEM, the BMSC‐exos were round or oval vesicles with double‐membraned structure, even size and diameter of 30‐100 nm (Figure 1E). It was found in NTA that the exosome diameter mainly was 87 nm, ranging 95.4 ± 18.9 nm (Figure 1F). Results of Western blot analysis suggested that CD63 and Alix expression was positive (Figure 1G).

MiR‐206 expression in BMSCs of each group was assessed by RT‐qPCR, and we discovered that miR‐206 mimic elevated miR‐206 expression, while miR‐206 inhibitor reduced miR‐206 expression (Figure 1H).

Under a microscope, osteoblasts were fusiform or polygonal‐shaped with different length of processes. Nuclei were round or oval with clear outline and situated at the centre of cells. (Figure 1I). As identified by ALP staining, the nuclei of primary osteoblasts were dark blue‐violet, cytomembrane and cytoplasm were light blue‐violet and many brown particles were observed in the cytoplasm (Figure 1J). The positive result of ALP staining indicated the ALP enrichment in cytoplasm.

### 3.2 miR‐206 in exosomes derived from BMSCs alleviates OA in mice and promotes proliferation and differentiation of osteoblasts

MiRNA has been revealed to be involved in the treatment of OA and other diseases. miR‐206 can regulate IGF‐1 in chondrocyte autophagy and apoptosis of OA rats.^18^ Moreover, miR‐206/cyclin D1 (CCND1) axis is involved in chondrocyte growth during the occurrence and development of OA.^19^ Elf3 is a transcription factor induced by inflammatory factors in various cell types including chondrocytes. A study has shown that the expression level of Elf3 in cartilage of patients with OA is increased.^22^ Here, we detected the expression of IGF‐1, CCND1 and Elf3 using RT‐qPCR and finally selected Elf3 with the largest difference in mRNA expression level as the research object (Supplementary Figure 1A,B).

**FIGURE 2:**
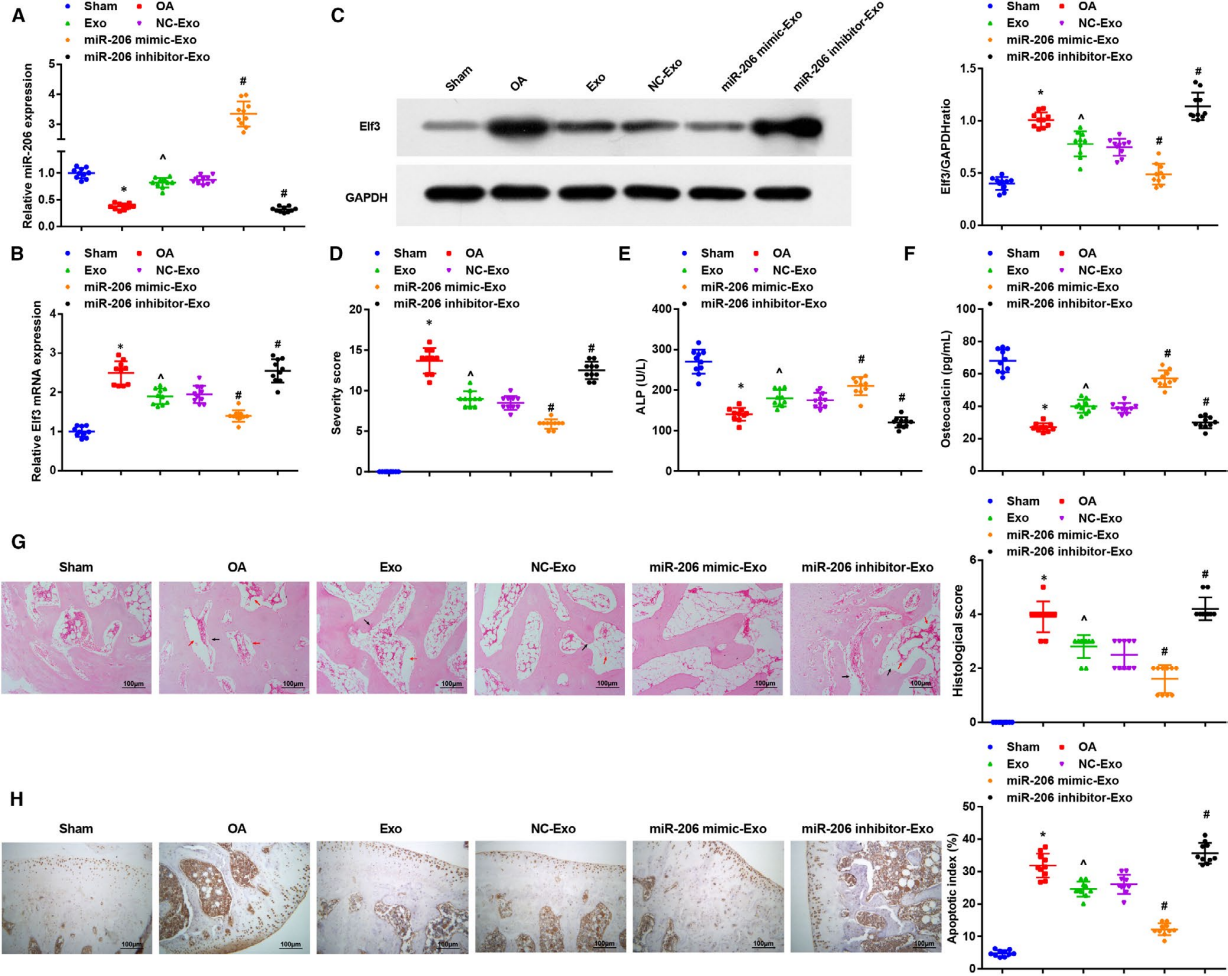
miR‐206 in exosomes derived from BMSCs alleviates OA in mice and promotes proliferation and differentiation of osteoblasts. A, expression of miR‐206 in mouse femoral tissues was determined by RT‐qPCR; B, expression of Elf3 in mouse femoral tissues was determined by RT‐qPCR; C, protein expression of Elf3 in mouse femoral tissues was determined by Western blot analysis; D, clinical OA scores of mice in each group; E, serum activity of ALP was evaluated by ELISA; F, serum content of OCN was measured by ELISA; G, representative images of HE staining and histopathological scores of mice in each group (red arrows indicated voids in bone tissue, nucleolar deformation and fusion; black arrows indicated thinning and fractured trabecular bone); H, osteoblast apoptosis in mouse bone tissues was assessed by TUNEL staining; n = 10; *, *P* < .05 vs the sham group, ^, *P* < .05 vs the OA group, #, *P* < .05 vs the NC‐Exo group; the measurement data were expressed as mean ±standard deviation, one‐way ANOVA was used for comparisons among multiple groups, and Tukey's post hoc test was used for pairwise comparisons after one‐way ANOVA

Expression levels of miR‐206 and Elf3 in mouse femoral tissues were determined. It came out that relative to the femoral tissues from sham‐operated mice, miR‐206 was down‐regulated while Elf3 was up‐regulated in OA mouse femoral tissues; BMSC‐exos increased miR‐206 expression while decreased Elf3 expression, and miR‐206 in BMSC‐exos elevating miR‐206 further up‐regulated miR‐206 and down‐regulated Elf3; results induced by BMSC‐exos inhibiting miR‐206 were opposite to that induced by BMSC‐exos elevating miR‐206 (Figure 2A‐C).

Clinical scores of mice and the serum levels of OCN and ALP were evaluated. We found that the OA mice had increased mean clinical score and decreased levels of OCN and ALP versus sham‐operated ones; BMSC‐exos reduced the mean clinical score and increased levels of OCN and ALP; the treatment of BMSC‐exos up‐regulating miR‐206 further reduced the mean clinical score and increased levels of OCN and ALP; the BMSC‐exos down‐regulating miR‐206 had totally opposite effects on OA mice (Figure 2D‐F).

As observed through HE staining and histopathological scoring, there were normal osteoblasts without shrinkage of cellular membrane in mouse femur of the sham‐operated mice; in the OA mice and OA mice treated with BMSC‐exos inhibiting miR‐206, there were abundant empty lacunae, nucleolar deformation and pyknosis in remaining osteocytes, decreased osteoblasts and thinned and fractured trabecula; in the OA mice treated with BMSC‐exos elevating miR‐206, there were osteocytes with normal morphology, newborn and thickened trabecula and a small amount of empty lacuna. In the OA mice treated with BMSC‐exos and BMSC‐Exo NC, there were lateralized osteocytes, karyopyknosis, partial empty lacuna, relatively less osteoblasts, osteoclasts and relatively thinned trabecula. The histopathological score of the OA group was higher than that of the sham group, and the histopathological score of the Exo group was lower than that of the OA group; in relation to the mice treated with BMSC‐Exo NC, mice treated with BMSC‐exos elevating miR‐206 had lower histopathological score while mice in the BMSC‐exos down‐regulating miR‐206 had higher histopathological score (Figure 2G).

In TUNEL staining, the positive cells had brown‐ or brownish yellow‐stained nuclei, and positive particles appeared in cytoplasm of several cells. Marrow tissues and osteocytes in trabecula in the sham group had some scattered apoptotic cells, and the osteocyte apoptosis was not obvious. Relative to the sham‐operated mice, the OA mice had an increased apoptotic index (AI); the increased AI in OA mice was reduced by BMSC‐exos and further reduced by BMSC‐exos elevating miR‐206; the BMSC‐exos inhibiting miR‐206 had a higher AI (Figure 2H).

### 3.3 miR‐206 in exosomes derived from BMSCs relieves inflammation and promotes osteoblast differentiation

**FIGURE 3:**
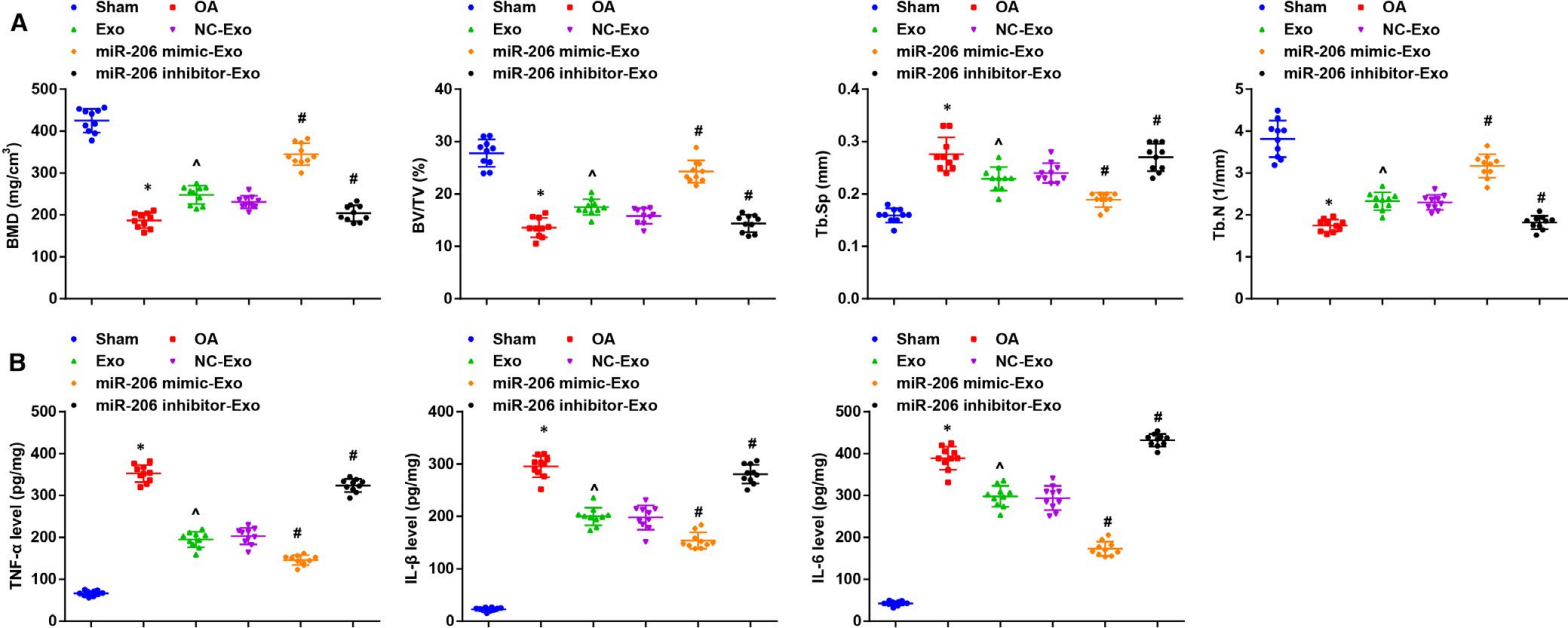
miR‐206 in exosomes derived from BMSCs relieves inflammation and promotes osteoblast differentiation. A, BMD, BV/TV, Tb. Sp and Tb.N in mouse bone tissues were measured by micro‐CT examination; B, contents of TNF‐α, IL‐1β and IL‐6 in mouse bone tissues were evaluated using ELISA; n = 10; *, *P* < .05 vs the sham group, ^, *P* < .05 vs the OA group, #, *P* < .05 vs the NC‐Exo group; the measurement data were expressed as mean ±standard deviation, one‐way ANOVA was used for comparisons among multiple groups, and Tukey's post hoc test was used for pairwise comparisons after one‐way ANOVA

BMD, BV/TV, Tb. Sp and Tb.N were analysed by micro‐CT and we found that BMD, BV/TV and Tb.N were decreased while Tb. Sp was increased in OA mice versus the sham‐operated ones; the treatment of BMSC‐exos or BMSC‐exos overexpressing miR‐206 enhanced BMD, BV/TV and Tb.N and restrained Tb. Sp; BMD, BV/TV and Tb.N were decreased while Tb. Sp was increased by BMSC‐exos reducing miR‐206 (Figure 3A).

Inflammatory factors in mouse bone tissues were measured using ELISA. The results reflected that contents of TNF‐α, IL‐1β and IL‐6 in the OA mice were all higher than that in the sham‐operated mice; treatment of BMSC‐exos or BMSC‐exos up‐regulating miR‐206 suppressed contents of TNF‐α, IL‐1β and IL‐6; treatment of BMSC‐exos down‐regulating miR‐206 increased the contents of TNF‐α, IL‐1β and IL‐6 in OA mice (Figure 3B).

### 3.4 miR‐206 in exosomes derived from BMSCs promotes proliferation and differentiation and inhibits apoptosis of osteoblasts from OA mice

**FIGURE 4:**
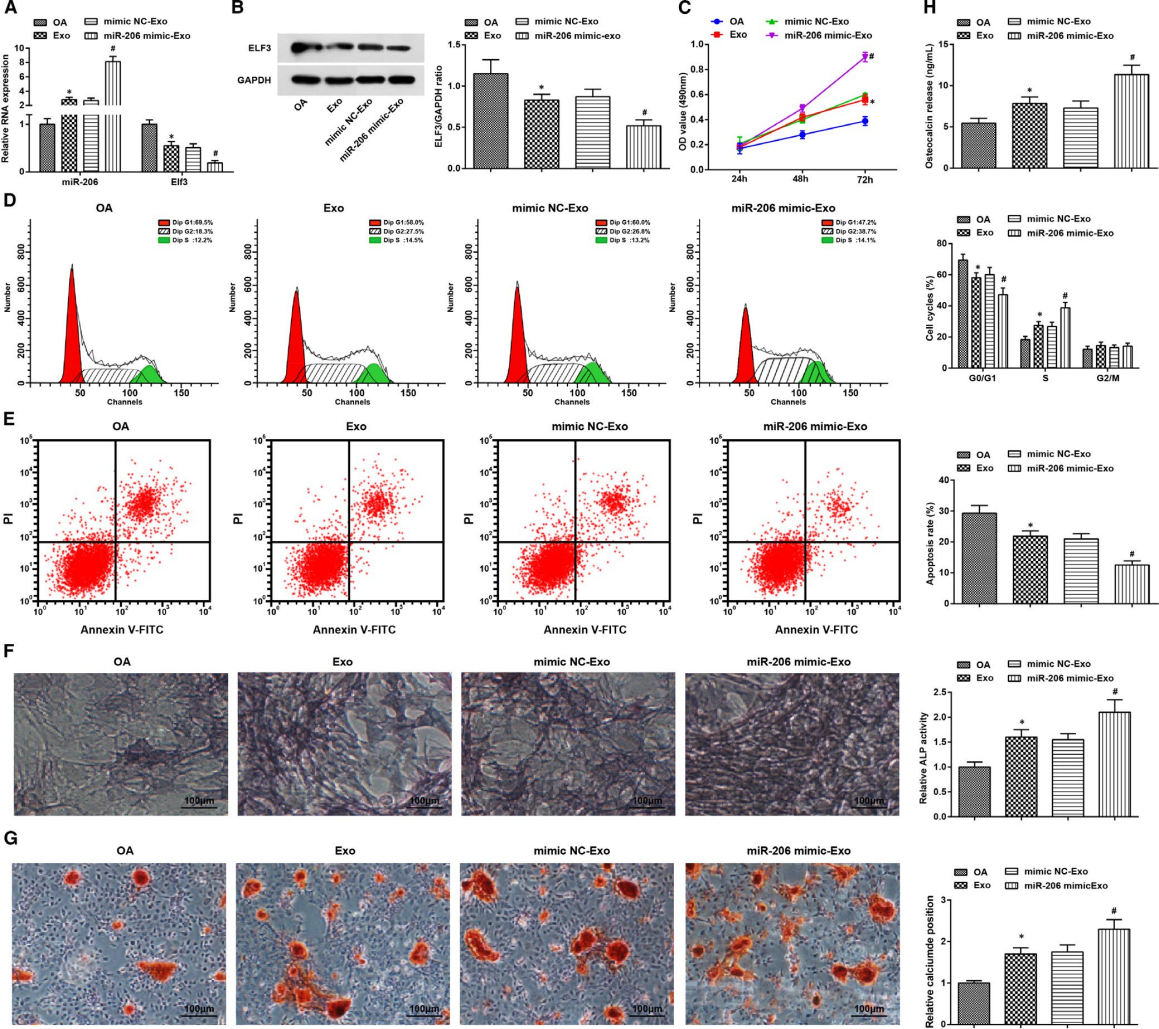
miR‐206 in exosomes derived from BMSCs promotes proliferation and differentiation and inhibits apoptosis of osteoblasts from OA mice. A, expression of miR‐206 and Elf3 in osteoblast was assessed by RT‐qPCR; B, protein expression of Elf3 in osteoblast was assessed using Western blot analysis; C, osteoblast proliferation was gauged by MTT assay; D, cell cycle distribution of osteoblasts were evaluated by flow cytometry; E, osteoblast apoptosis was evaluated by flow cytometry; F, ALP activity in osteoblasts was assessed using ALP staining; G, calcium deposition level of osteoblasts was determined by alizarin red staining; H, OCN secretion in osteoblasts was assessed by radioimmunoassay; N = 3; *, *P* < .05 vs the OA group, #, *P* < .05 vs the mimic NC‐Exo group; the measurement data were expressed as mean ±standard deviation, one‐way ANOVA was used for comparisons among multiple groups, and Tukey's post hoc test was used for pairwise comparisons after one‐way ANOVA

Expression levels of miR‐206 and Elf3 in osteoblasts in each group were gauged. The outcome indicated that miR‐206 was up‐regulated while Elf3 was down‐regulated after treatment of BMSC‐exos or BMSC‐exos conveying miR‐206 mimic (Figure 4A,B).

Results of MTT assay and flow cytometry revealed that BMSC‐exos or BMSC‐exos up‐regulating miR‐206 increased cell viability and cells in the S phase and decreased the apoptosis rate and cells in the G0/G1 phase (Figure 4C‐E).

It was observed through ALP staining, alizarin red staining and radioimmunoassay that BMSC‐exos enhanced ALP activity, calcium deposition level and OCN secretion, which were further increased by treatment of BMSC‐exos up‐regulating miR‐206 (Figure 4F‐H).

### 3.5 MiR‐206 targets Elf3

**FIGURE 5:**
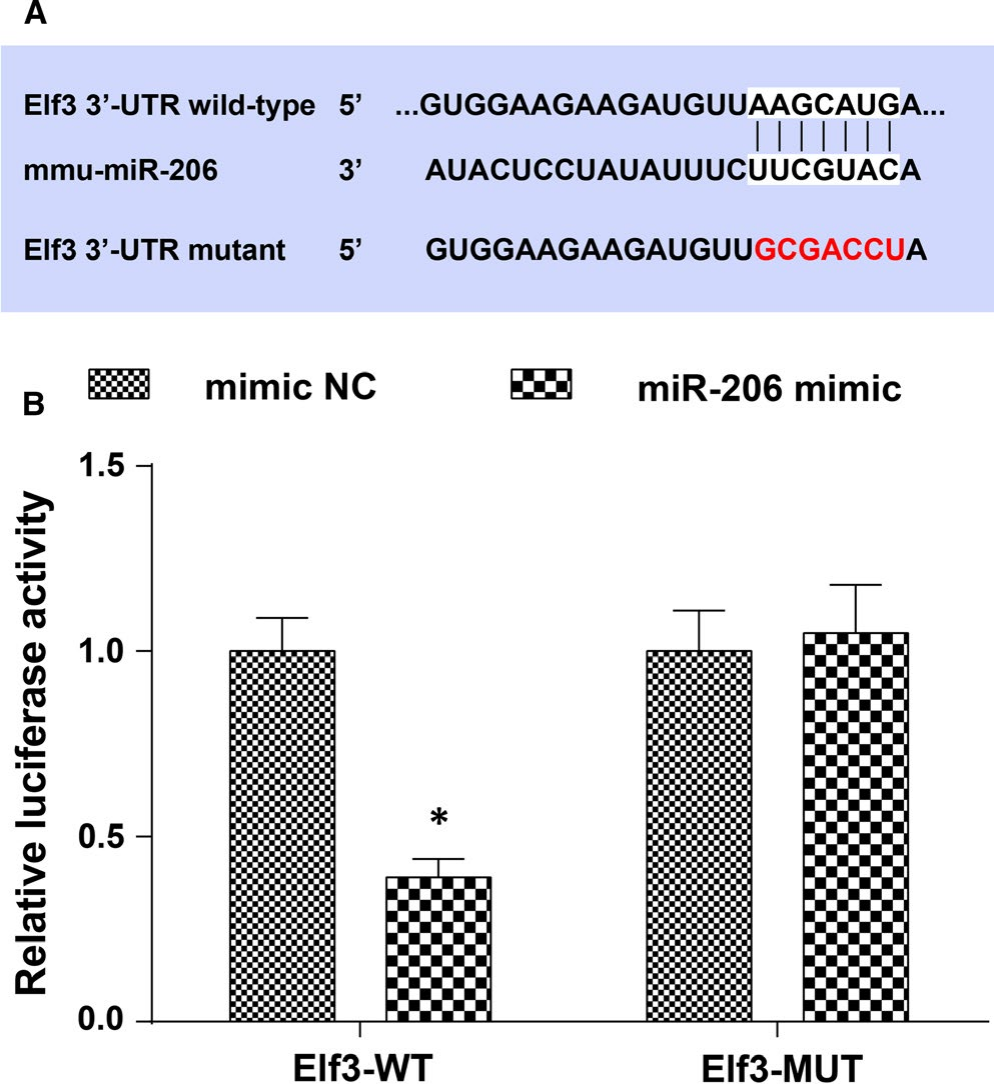
MiR‐206 targets Elf3. A, a bioinformatic website was applied to predict the target site of miR‐206 and Elf3, and the mutant sequence of Elf3; B, target relation between miR‐206 and Elf3 was confirmed by dual luciferase report gene assay; N = 3, *, *P* < .05 vs the mimic NC group; the measurement data were expressed as mean ±standard deviation, and unpaired t test was performed for comparisons between two groups

It was predicted by a bioinformatic website that there existed binding sites between miR‐206 and Elf3 (Figure 5A). Furthermore, we found through the dual luciferase reporter gene assay that the co‐transfection of WT Elf3 and miR‐206 mimic into 239T cells suppressed the luciferase activity, indicating that miR‐206 was able to target Elf3 (Figure 5B).

### 3.6 Elevated miR‐206 or inhibited Elf3 promotes proliferation and differentiation and inhibits apoptosis of osteoblasts from OA mice

**FIGURE 6:**
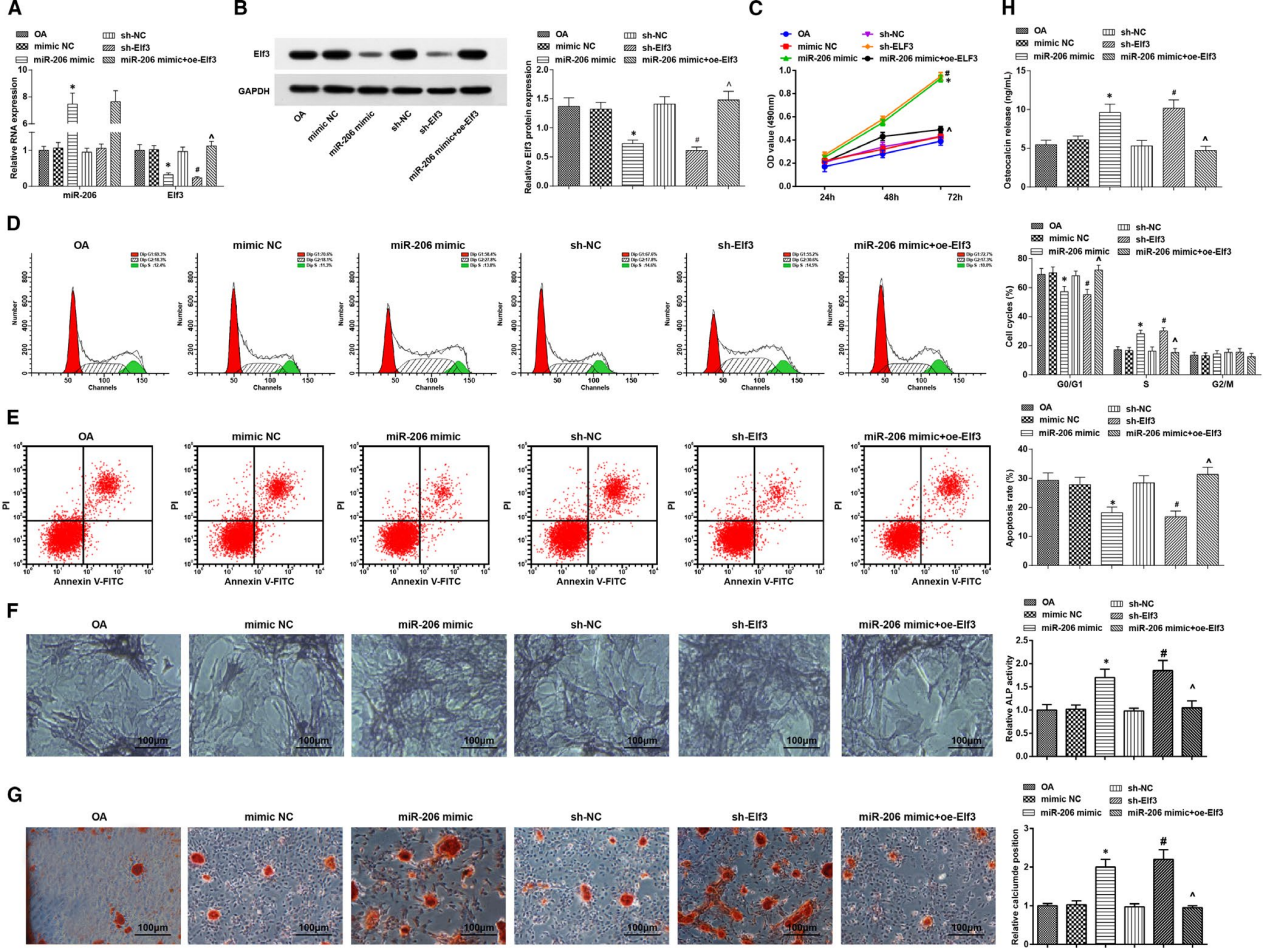
Elevated miR‐206 or inhibited Elf3 promotes proliferation and differentiation and inhibits apoptosis of osteoblasts from OA mice. A, expression of miR‐206 and Elf3 in osteoblast was assessed by RT‐qPCR; B, protein expression of Elf3 in osteoblast was assessed using Western blot analysis; C, osteoblast proliferation was gauged by MTT assay; D, cell cycle distribution of osteoblasts was evaluated by flow cytometry; E, osteoblast apoptosis was determined using flow cytometry; F, ALP activity in osteoblasts was assessed using ALP staining; G, calcium deposition level of osteoblasts was determined by alizarin red staining; H, OCN secretion in osteoblasts was assessed by radioimmunoassay; N = 3; *, *P* < .05 vs the mimic NC group, #, *P* < .05 vs the sh‐NC group, ^, *P* < .05 vs the miR‐206 mimic group; the measurement data were expressed as mean ±standard deviation, one‐way ANOVA was used for comparisons among multiple groups, and Tukey's post hoc test was used for pairwise comparisons after one‐way ANOVA

MiR‐206 and Elf3 expression in osteoblasts in each group was assessed, and the outcome implied that miR‐206 was up‐regulated while Elf3 was down‐regulated after miR‐206 elevation; treatment of silencing Elf3 reduced Elf3 expression; the decreased Elf3 expression that induced by miR‐206 elevation was reversed by Elf3 overexpression (Figure 6A,B).

Osteoblast proliferation, cell cycle arrest and apoptosis were determined. It was discovered that cell viability and cells in the S phase were increased, and the apoptosis rate and cells in the G0/G1 phase were decreased after treatment of miR‐206 up‐regulation or Elf3 knockdown; overexpression of Elf3 reversed miR‐206 up‐regulation‐induced effects (Figure 6C‐E).

ALP activity, calcium deposition level and OCN secretion were evaluated, and it came out that elevated miR‐206 or inhibited Elf3 enhanced ALP activity, calcium deposition level and OCN secretion; the effects of elevated miR‐206 on ALP activity, calcium deposition level and OCN secretion in OA osteoblasts were abrogated by Elf3 overexpression (Figure 6F‐H).

## 4 DISCUSSION

OA is known as a progressive degeneration of the articular cartilage, and the subchondral bone osteoblast metabolism is abnormally affected in OA.^28^ miR‐206 is involved in the process of OA,^15^ while BMSCs can deliver a large amount of miR‐206,^14^ so the use of BMSC‐exos can be better than the direct use of miR‐206. Given that, the present study explored the impact of BMSC‐derived exosomal miR‐206 in the osteoblast proliferation and differentiation during the development of OA with the involvement of Elf3. We have confirmed that the exosomal miR‐206 from BMSCs promoted proliferation and differentiation of osteoblasts in OA through reducing Elf3.

We constructed animal and cellular models to observe the effect of BMSC‐exos, exosomal miR‐206 and Elf3 on OA progression. It was found that the treatment of BMSC‐exos ameliorated inflammatory response and osteoblast proliferation and differentiation and reduced osteoblast apoptosis in OA. Similarly, Li *et al* have found that BMSC‐exos attenuated inflammation of the central nervous system.^29^ The injection of BMSC‐exos into the necrosis region has been unravelled to promote bone regeneration and angiogenesis, thus decelerating the progression of steroid‐induced avascular necrosis of femoral head.^30^ Moreover, Liao *et al* have unearthed that BMSC‐exos promoted proliferation of osteoblasts in osteonecrosis of the femoral head,^31^ and a recent study has indicated that BMSC‐exos enhanced viability and restrain apoptosis of cardiomyocytes in myocardial ischaemia reperfusion injury.^32^ A recent document has also suggested that MSC‐derived exosomes promoted proliferation and inhibited apoptosis of chondrocytes, thereby decelerating the progression of OA.^15^ Moreover, we assessed the expression of miR‐206 in OA mouse femur and OA osteoblasts, and it was found that modelled mouse femur and osteoblasts showed a decreased expression level of miR‐206. In line with this finding, Cao *et al* have affirmed that miR‐206 was down‐regulated in OA cartilage tissues,^19^ and the down‐regulation has also been verified in dermatomyositis.^33^ The expression of miR‐206 was measured after treated with BMSC‐exos, and it was revealed that BMSC‐exos were able to elevate miR‐206 expression. A similar result has been found by Guescini *et al* that muscle tissue–released extracellular vesicles enriched miR‐206.^34^ Additionally, the role of BMSC‐exos conveying miR‐206 in OA models was observed in our study and we discovered that the up‐regulation of exosomal miR‐206 from BMSCs suppressed inflammatory response and osteoblast proliferation and differentiation and suppressed osteoblast apoptosis in OA. Consistently, a research has identified that the overexpression of miR‐206 alleviated chronic constriction injury‐triggered neuroinflammation in rats,^35^ and it has been figured out that Freund's adjuvant–induced inflammation in a mouse pain model was accompanied by the decreased expression of miR‐206 in a time‐dependent manner.^36^ Additionally, Yu *et al* have provided evidence that the elevation of miR‐206 promoted proliferation and repressed apoptosis of chondrocytes during the development of OA.^18^

In addition, the expression of Elf3 and the target relationship between miR‐206 and Elf3 was assessed in our study, and it came out that Elf3 was overexpressed and acted as a target gene of miR‐206 in OA models. Similar to this result, Wondimu *et al* have elucidated that the expression level of Elf3 was elevated in human cartilage from patients with OA,^22^ while the target relation between miR‐206 and Elf3 remains scarcely investigated. Similar to the elevated miR‐206, the inhibition of Elf3 was able to restrict inflammation and osteoblast proliferation and differentiation and repress osteoblast apoptosis in OA. In accordance with this outcome, a recent literature has revealed that Elf3 participated in cartilage destruction in OA as an inflammatory mediator, and the leptin synergized with IL‐1β to induce Elf3 expression in chondrocytes, thus triggering inflammatory response in chondrocytes.^37^ Besides, Oliver *et al* have discovered that in the peribronchiolar interstitium, the cell proliferation and mitosis were markedly promoted in Elf3^‐/‐^ mice than in Elf3^+/+^ mice.^38^

To sum up, we found that exosomal miR‐206 from BMSCs promoted the proliferation and differentiation of osteoblasts in OA through reducing Elf3, thereby decelerating the development of OA. Our study may contribute to exploration on OA treatment, while more endeavours remain to be done.

## CONFLICT OF INTEREST

The authors declare that they have no conflicts of interest.

## AUTHOR CONTRIBUTION

**Yijiang Huang:** Data curation (equal); Writing‐original draft (equal). **Xiumeng Zhang:** Data curation (equal). **Jingdi Zhan:** Formal analysis (equal). **Zijiang Yan:** Formal analysis (equal). **Daosen Chen:** Formal analysis (equal). **Xinghe Xue:** Formal analysis (equal). **Xiaoyun Pan:** Conceptualization (equal).

## Supporting information

Fig S1

## Data Availability

Research data not be shared.
