# Supplementary figures and images for: Bone marrow mesenchymal stem cell–derived exosomal miR‐206 promotes osteoblast proliferation and differentiation in osteoarthritis by reducing Elf3
Source: J Cell Mol Med. 2021 Jun 23;25(16):7734–45. doi: 10.1111/jcmm.16654 (PMC8358849; doi:10.1111/jcmm.16654)

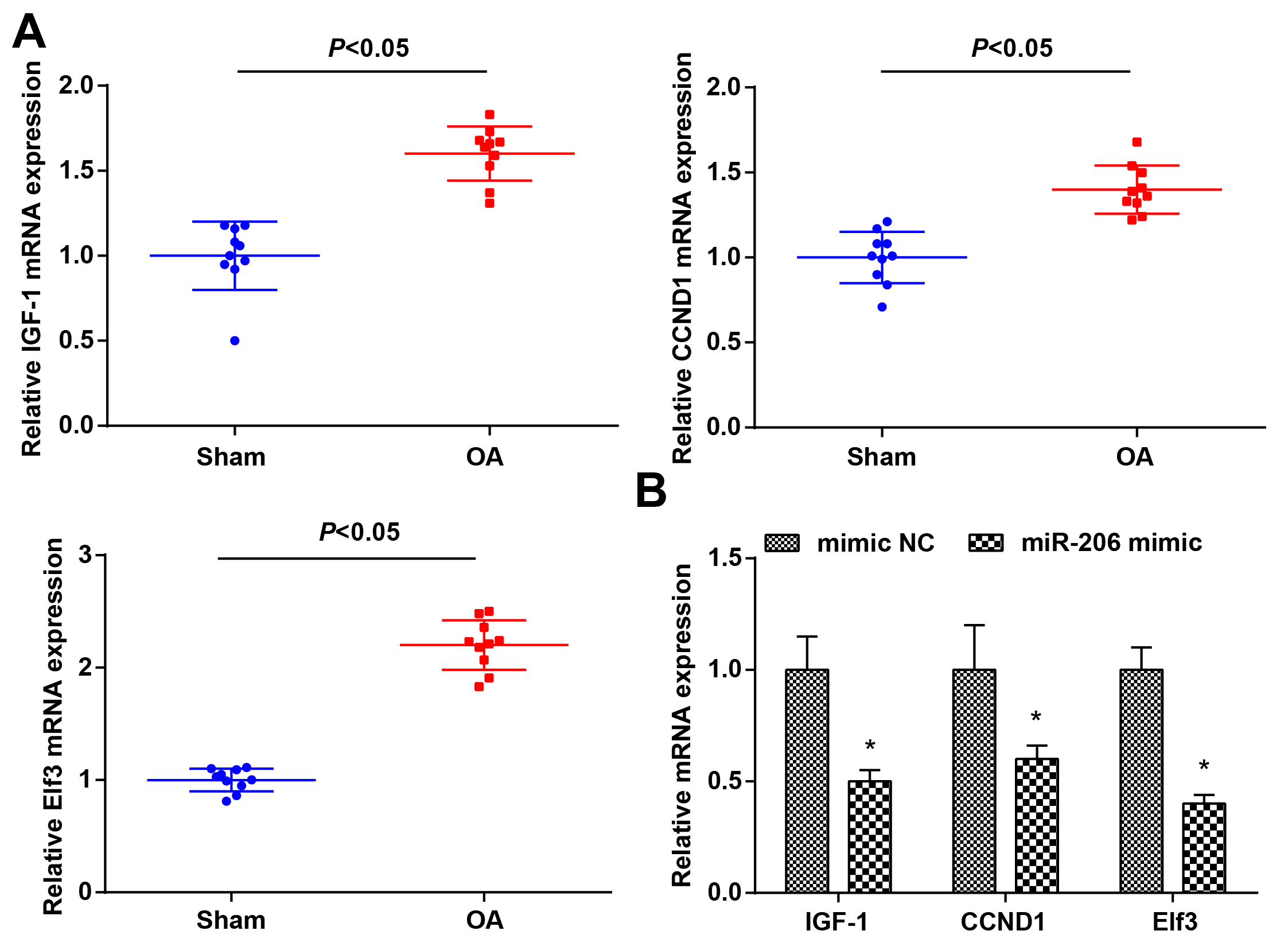

Supplement: Supplementary file 1 — Fig S1 [file JCMM-25-7734-s001.jpg]
